# Editorial: Rising stars in electrochemistry 2021

**DOI:** 10.3389/fchem.2022.1099546

**Published:** 2022-11-30

**Authors:** Valentín Briega-Martos, Jafar Soleymani, José H. Zagal

**Affiliations:** ^1^ Forschungszentrum Jülich GmbH, Helmholtz Institute Erlangen-Nürnberg for Renewable Energy (IEK-11), Erlangen, Germany; ^2^ Pharmaceutical Analysis Research Center, Tabriz University of Medical Sciences, Tabriz, Iran; ^3^ Laboratory of Electrocatalysis, Department of Chemistry of Materials, Faculty of Chemistry and Biology, University of Santiago de Chile, Santiago, Chile

**Keywords:** electrochemistry, biosensors, signal amplification, lithium-oxygen batteries, lithium-ion battery (LIB), polymer electrolytes, open-circuit potential, surface charge

As editors of the Research Topic “Rising stars in Electrochemistry 2021”, it is our pleasure to give an overview of the perspective, mini-review and original contribution articles included in the present issue. In this editorial we will sum up the main results and perspectives that are presented in the published papers.

Zhou et al. overviewed in their Perspective paper the recent advances in the different signal amplification strategies employed in bioelectrochemical sensors for infectious disease diagnosis. Considering the recent corona-virus pandemics, which brought up the great importance of virus detection, this Perspective paper is of special interest. The infectious disease diagnosis can be performed using electrochemical techniques providing fast and reliable methods for the detection of several infections. The amplification strategies can be classified, on the one hand, as target-based amplification, which increases the abundance of the targeted analyte, and, on the other hand, signal-based amplification, which decreases the limit of detection (LOD) by modifying the transducer system to improve the signal-to-noise ratio. Despite the promising advances detailed in this paper, more is needed to overcome the drawbacks of this type of sensors, such as the use of expensive and specialized reagents or the necessity of multiple signal amplification techniques or a combination of materials that can increase the cost-per-test and the time for the result. Therefore, in addition to improving the LOD, future research strategies should aim for the detection of the minimal infectious dose of the target disease, decrease the cost-per-test and pre-analyze steps, and promote ease of operation. Also, the development of the point-of-care (PoC) method for preliminary screening of infectious diseases is another aim that must be regarded in future research strategies.

Lithium batteries have come to play a central role as power sources of computers and portable phones and might play a major role in electromobility in the near future. Two articles describe recent advances in this field.

In the Mini-Review by Zhang et al., the last progress made on Li-O_2_ batteries based on LiOH formation and decomposition, giving special attention to the reaction mechanisms that occur in the cathode and the stability of the cathode binder and Li anode, is presented. Lithium-oxygen (Li-O_2_) batteries use Li metal as the anode and an oxygen reduction active material on the cathode, and they can be divided into aqueous or non-aqueous Li-O_2_ batteries, the latter being the most commonly studied. Within the non-aqueous type, Li-O_2_ batteries based on LiOH chemistry can be operated in humid environments and demonstrate better resistance towards CO_2_ than Li_2_-O_2_ batteries, the non-aqueous battery most extensively investigated. After presenting all the most recent advances in the field of Li-O_2_ based on LiOH chemistry, the authors conclude that the next research lines should focus on systematic studies of the reaction mechanisms with advanced characterization techniques such as Raman spectroscopy or isotope-labelled differential electrochemical mass spectrometry (DEMS) in combination with theoretical calculations, the production of new electrocatalysts that can mitigate the detrimental reactive intermediates such as surface hydroxyl radicals, and the development of Li anode protection technologies.

In the work by Sai et al. the authors present a systematic study by thermal, electrochemical and spectroscopic techniques of the electrolyte properties of some lithium air batteries (LIBs). They study a single polyether having alkyl side chains with varied lengths for its application as polymer electrolyte for LIBs since polymer structure is crucial for promoting lithium-ion conduction and improving cell performance. The influence of the alkyl side chain on the coordination structure around the lithium-ion and ether group and the lithium-ion transport properties for the polyether electrolytes is investigated. The results show that the length of the alkyl side chain affects the lithium coordination structure. The alkyl side chain also has a plasticizing effect that increases the segmental polymer mobility. In addition, as the contribution from the non-polar alkyl side chain increases, the overall dielectric constant of the electrolyte and the LiTFSA dissociability decrease. Finally, they find that the transference number of lithium-ion increased with the extension of the alkyl side chain. This is attributed to the effective inhibition of the transport of the TFSA anion because of the steric hindrance of the alkyl side chain. This work points out the importance of properly selecting the length of the alkyl side chain to improve the electrochemical properties of the polymer electrolyte.

Lastly, Huang and Zhang present on their paper a revision of the concept of open-circuit voltage of an electrochemical cell and its relation to charge distribution at the electrode-electrolyte interface. The authors derive an equilibrium Poisson-Nernst equation that describes how the charge distribution across the electrical double layer can be dictated by the electrochemical potential of the redox species in these conditions. With the example of a H_2_/O_2_ fuel cell it is shown that the OCV originates microscopically *via* the spillover electron in the electrical double layer. The authors finally note the difference between the potential of zero charge (pzc) that is formed in these OCV conditions and that it can be very different when comparing anode and cathode of the same material, and the traditional concept of pzc used for comparing with the work function of the metal and that is usually investigated in closed-circuit conditions. It is important to point out that a electrochemical cell delivering energy is not at equilibrium and the cell voltage is lower that the thermodynamic potential of that particular cell.

